# An Abrupt Doubling of Heart Rate

**DOI:** 10.1016/j.jaccas.2026.109304

**Published:** 2026-07-29

**Authors:** Mazen M. Kawji

**Affiliations:** Cardiovascular Institute, Order of Saint Francis, Ottawa, Illinois, USA

**Keywords:** atrial fibrillation, bradycardia, electrocardiogram

## Abstract

**Case Summary:**

Sinoatrial exit block is one manifestation of sick sinus syndrome. The impulse generated by the sinus node either fails to depolarize the atria or does so with delay. Sinoatrial exit block could vary in severity from first-, to second-, to third-degree. We here present a case of one type of sinoatrial block that is rarely encountered. The patient had atrial fibrillation and several other cardiac and medical comorbidities, which may complicate management. The patient had other conduction abnormalities, but no evidence of atrioventricular disease.

**Take-Home Message:**

Doubling/halving of the heart rate should raise suspicion of 2:1 sinoatrial block, indicating sinus node dysfunction.

## Graphical Abstract

**Figure:**
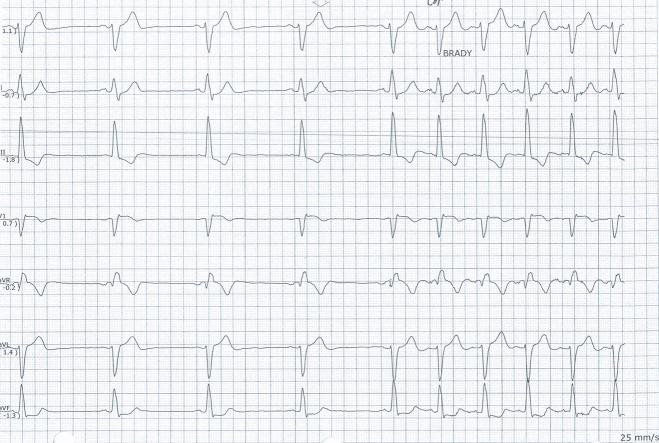

## Case Presentation

**Figure 1:**
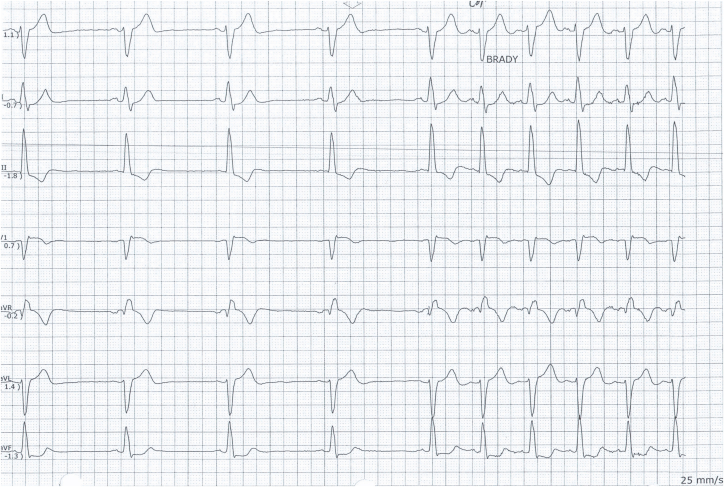
A Rhythm Strip Showing an Abrupt Doubling of the Patient's Heart Rate

An 86-year-old white male patient was admitted to our hospital with shortness of breath. He had severe anemia due to gastrointestinal bleeding. The patient had a history of coronary artery bypass surgery, paroxysmal atrial fibrillation, diastolic congestive heart failure, and transcatheter aortic valve replacement. His medications included apixaban and metoprolol. The telemetry technician noticed a change in the patient's heart rate and recorded a rhythm strip (Figure 1). The patient was resting comfortably in bed without any complaints. What is the cause of doubling of the heart rate?

1. Resolution of nonconducted, bigeminal premature atrial contractions.
2. 2:1 sinoatrial block.
3. There is no change in heart rate. The speed of recording was decreased from 50 mm/s to 25 mm/s.
4. Can't tell. Need to acquire a 12-lead electrocardiogram (ECG).

## Response to ECG Challenge

Resolution of 2:1 sinoatrial block is the correct answer. The rhythm strip (Figure 1) shows severe sinus bradycardia, followed by an abrupt change to normal sinus rhythm; with doubling of the patient's heart rate from 40 to 79 to 82 beats/min. The PP interval in the first half of the rhythm strip is about 1,510 milliseconds. In the second half of the rhythm strip, the first PP interval is 760 milliseconds (almost half 1,510 milliseconds), then slightly gets shorter due to sinus arrhythmia.

Left bundle branch block and extreme right axis deviation are noted and confirmed by a 12-lead ECG (not shown). Left bundle branch block is new, whereas right axis deviation is old. The P-wave, the QRS complex, and the PR interval all remain the same.

There is no evidence of blocked bigeminal premature atrial contractions. The 7-lead rhythm strip shows no deformity in the T-wave to suspect a superimposed P wave due to a blocked premature atrial contraction.

A change in the paper's speed would have led to a change in the width of QRS complexes, which is not the case.

Pacemaker cells in the sinus node generate electrical impulses that initiate the heart rhythm. Sinoatrial conduction is the time it takes for an electrical impulse to travel from the sinus node to the surrounding atrial tissue. Its duration is 45 to 125 milliseconds. In case of sinoatrial block, the impulse generated by the sinus node either fails to depolarize the atria or does so with delay. There are 3 degrees of sinoatrial blocks. First-degree sinoatrial exit block occurs when sinus node impulse reaches the atrial tissue after a delay; it cannot be detected on an ECG or on rhythm strips. Sinus node electrogram recording is needed.

Type 1 second-degree (Wenckebach-type) sinoatrial exit block is diagnosed when there is a progressive shortening of the P-P interval, followed by a pause that is less than twice the preceding P-P interval. It is sometimes difficult to differentiate it from sinus arrhythmia.^1^^,^^2^

**Figure 2:**
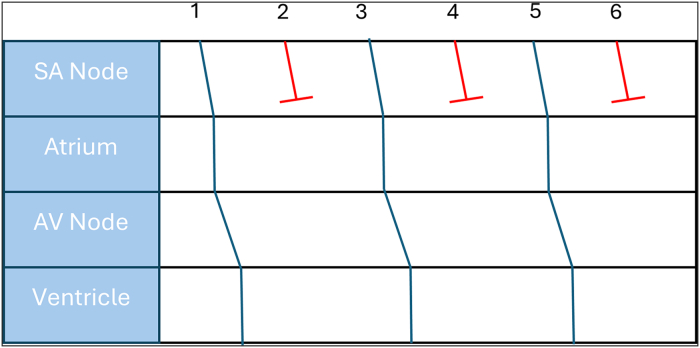
A Laddergram Explaining 2:1 SA Block Numbers 1 to 6 represent sinus node discharges. The first beat shows a sinus node discharge reaching the atrial tissue after a physiologic delay (sinoatrial [SA] conduction time), discharging the atrium. After an atrioventricular (AV) node delay, the impulse depolarizes the ventricle. The next sinus node discharge is blocked and never reaches the atrium. There is no PQRST complex. It is difficult to differentiate 2:1 SA block from sinus bradycardia.

Type 2 second-degree sinoatrial exit block is easily diagnosed by detecting a pause that is twice the duration of the basic P-P interval, in the absence of a blocked premature atrial beat. Then there is type 2, 2:1 second-degree sinoatrial exit block, in which every other sinus node impulse fails to depolarize the atria, an example of which is presented here. This results in halving of the baseline sinus rate (see laddergram in Figure 2). Third-degree/high-grade sinoatrial exit block is suspected when prolonged pauses are noted (without P waves) that are several times the duration of the P-P interval. However, the presence of an escape rhythm may mask this type of sinoatrial exit block. In contrast, the pause of sinus arrest has no mathematical relationship to the basic sinus cycle (ie, not multiple of the shortest P-P interval). Sinoatrial block is a form of sinus node dysfunction. Sinus node dysfunction may coexist with atrioventricular nodal disease. Our patient had evidence of conduction system disease as well.

Patients with sinoatrial exit block frequently have additional atrial arrhythmias such as paroxysmal atrial fibrillation. This was the case in our patient. Sinoatrial exit block may result from degenerative sinus node disease, drug toxicity, acute myocardial infarction, acute myocarditis (including Lyme carditis, which may afflict young patients; conduction disease could be reversible with appropriate antibiotic therapy), rheumatic heart disease, and infiltrative cardiomyopathies. It is less likely to be seen in healthy individuals due to increased vagal tone.^3^ Symptomatic sinus node dysfunction is a class 1 indication for atrial-based pacing according to the 2018 American College of Cardiology/American Heart Association/Heart Rhythm Society guidelines^4^ (sinoatrial block was addressed under sinus node dysfunction). Pacing will enable control of frequently accompanying tachyarrhythmias. A 2:1 sinoatrial exit block is a rarely encountered arrhythmia.

The patient received 50 mg of an extended-release metoprolol 18 hours before the rhythm strip was recorded at 5 am. Left bundle branch block was a new finding. The combination of right axis deviation and left bundle branch block is rare. Most commonly it is seen in severe cardiomyopathy with biventricular enlargement. The persistence of right axis deviation after the development of left bundle branch block is more consistent with biventricular enlargement than the presence of left posterior fascicular block. The shortness of breath was attributed to severe anemia. Apixaban was held. The patient had no history of chronic lung disease. Overall, it appeared the patient had progressive conduction disease at several levels. He was eventually discharged in stable condition.

## Funding Support and Author Disclosures

The author has reported that he has no relationships relevant to the contents of this paper to disclose.

**Take-Home Messages**

• Doubling/halving of the heart rate should raise suspicion of 2:1 sinoatrial block, indicating sinus node dysfunction.
• Atrial-based pacing should be considered in patients with severe, symptomatic sinus node dysfunction.
